# The role of action planning and plan enactment for smoking cessation

**DOI:** 10.1186/1471-2458-13-393

**Published:** 2013-04-26

**Authors:** Hein de Vries, Sander M Eggers, Catherine Bolman

**Affiliations:** 1 Care and Public Health Research Institute, Department of Health Promotion, Maastricht University, Maastricht, The Netherlands; 2 Faculty of Psychology, Open University of the Netherlands, PO Box 2960, Heerlen 6401 DL, The Netherlands

**Keywords:** Smoking cessation, Action planning, Preparatory planning, Plan enactment, Planned quit attempts

## Abstract

**Background:**

Several studies have reemphasized the role of action planning. Yet, little attention has been paid to the role of plan enactment. This study assesses the determinants and the effects of action planning and plan enactment on smoking cessation.

**Methods:**

One thousand and five participants completed questionnaires at baseline and at follow-ups after one and six months. Factors queried were part of the I-Change model. Descriptive analyses were used to assess which plans were enacted the most. Multivariate linear regression analyses were used to assess whether the intention to quit smoking predicted action planning and plan enactment, and to assess which factors would predict quitting behavior. Subsequently, both multivariate and univariate regression analyses were used to assess which particular action plans would be most effective in predicting quitting behavior. Similar analyses were performed among a subsample of smokers prepared to quit within one month.

**Results:**

Smokers who intended to quit smoking within the next month had higher levels of action planning than those intending to quit within a year. Additional predictors of action planning were being older, being female, having relatively low levels of cigarette dependence, perceiving more positive and negative consequences of quitting, and having high self-efficacy toward quitting. Plan enactment was predicted by baseline intention to quit and levels of action planning. Regression analysis revealed that smoking cessation after six months was predicted by low levels of depression, having a non-smoking partner, the intention to quit within the next month, and plan enactment. Only 29% of the smokers who executed relatively few plans had quit smoking versus 59% of the smokers who executed many plans. The most effective preparatory plans for smoking cessation were removing all tobacco products from the house and choosing a specific date to quit.

**Conclusion:**

Making preparatory plans to quit smoking is important because it also predicts plan enactment, which is predictive of smoking cessation. Not all action plans were found to be predictive of smoking cessation. The effects of planning were not very much different between the total sample and smokers prepared to quit within one month.

## Background

Behavioral change strategies and pharmacotherapy are often recommended for smoking cessation because they have been found to be (cost)-effective [1]. Furthermore, a problem of behavior change in general, and thus also smoking cessation, is that people may indicate a willingness to change their behavior, but often fail to act on their intentions [2,3]. The intention-behavior gap is receiving increasing attention. One of the recommended strategies to overcome this gap pertains to goal setting; persons undertake specific actions in order to carry out their intentions. Translating an intention into behavior requires self-regulative strategies that entail both cognitive and enactive acts, strategies that we refer to as action planning [4-8]. The latter has been found to be an effective way of bridging the intention-behavior gap for both health and patient education [8-17].

Several descriptions of action planning are available [5,9,14,16-19]. The commonality shared by the various descriptions is that action planning implies a sequence of behaviors used to translate an individual’s intention into specific actions in order to achieve a desired behavior goal [20]. Action planning – as for instance outlined in the I-Change Model [8,21,22] – can be described as the process of planning and executing specific actions that may help to adopt successfully and maintain a new health behavior. Action planning thus refers to the process by which a person aims to translate intentions into the goal behavior by setting various detailed plans – action plans – to carry out and maintain this goal behavior [23]. Effective action planning is more likely to occur when persons are motivated to change [5,17,24].

Goal setting refers to the end-goal that one wishes to accomplish [25,26], and action planning refers to the choice of actions needed to achieve this goal and is comparable to setting proximal subgoals and using specific task strategies. Attainable action plans contribute to increased self-efficacy and task persistence, and will increase the likelihood of achieving the goal [27,28]. The beneficial effect of action planning is also acknowledged by Bagozzi [4,29], who discusses the importance of preparatory actions in the process of translating an intention into behavior [12]. The importance of action plans is also acknowledged by the Transtheoretical model by identifying various processes of change by which individuals seek new information and find alternatives for their unhealthy behavior [30]. In addition, action planning has similarities with performance objectives as described by Bartholomew and colleagues [31], which pertain to the exact performances (actions) a person needs in order to achieve a final learning objective.

Action planning can entail at least two types of actions: preparatory actions and coping actions. Preparatory actions are actions that a person needs to undertake to prepare the behavior-change attempt [16,32-34]. Coping actions refer to actions needed to cope with difficult situations that may endanger the maintenance of the newly acquired health behavior [13,14,17,35]. Planning preparatory and coping actions may require a substantial amount of self-regulative effort. This contrasts with a particular type of action plan that is referred to as implementation intentions. Implementation intentions require less self-regulation, because their formation consists of actions that will (almost) automatically be executed when a specified cue occurs [5]. Although the application of implementation intentions can lead to significant effects [36], some studies found no effects aof implementation intention interventions on behavior [37-40]. One explanation for this lack of effects may lie in the fact that intention formation outside the laboratory consists of self-chosen plans [14], which are not necessarily effective plans. In sum, action planning, as referred to in the I-Change model, encompasses a range of different types of planning strategies that may differ in their level of self-regulation.

With regard to smoking cessation, several recommendations for action plans can be found in the literature as well as cessation guides, such as: planning to remove all tobacco products from the house; planning to ask guests not to smoke inside; planning to inform friends that you are about to quit; planning how to cope with withdrawal symptoms; planning to remain smoke free when feeling stressed, sad, or down; planning how to remain smoke free when seeing others smoke or ashtrays (for more examples, see [41,42]). Yet, little research has been done to test the efficacy of these plans in the process of smoking cessation. Moreover, some plans may be more effective for quitting, but may not necessarily be instrumental in achieving long-term change. Not all plans (or strategies as they are also known) may be effective. For instance, switching to “light” cigarettes has been found to be ineffective [43]. Hoving and colleagues [41] as well as Scholz and colleagues [44] found that action planning was positively related to successful quitting. Van Osch and colleagues [16] demonstrated that the addition of making coping plans in a Quit and Win campaign led to significantly higher quit rates. Hilberink and colleagues [42] found that Chronic Obstructive Pulmonary Disease (COPD) patients who were motivated to quit smoking within the next six months made significantly more action plans than those planning to quit smoking within the next year [42]. Yet, these studies did not assess which particular plans were predictive for smoking cessation.

Intuitively, the most important aspect of choosing particular action plans lies in their successful enactment [45]. However, one study revealed that, when given the opportunity, smokers actually used very few action plans in their smoking cessation attempt [22]. In order to be able to motivate smokers to choose effective plans, we need to know the efficacy of specific plans. Although goal setting implies both action planning and plan enactment [6,12,26,31], little research is available describing the contributions of both planning and plan enactment.

This longitudinal study analyzed the role of action plans and their enactment in predicting attempts to quit and smoking cessation after one and six months. First, we describe which plans were chosen and which were enacted. Second, we examine which plans were related to attempts to quit and smoking cessation. Next, we test four hypotheses: Hypothesis 1, which is congruent with notions formulated by Sheeran, Webb, and Gollwitzer [24], states that individuals who intend to quit smoking within the next month will exhibit higher levels of planning than those intending to quit within a longer period of time. Hypothesis 2 states that higher levels of intention (Hypothesis 2a) and planning (Hypothesis 2b) will also lead to higher levels of plan enactment. Additionally, we postulate that higher levels of planning (Hypothesis 3) and plan enactment (Hypothesis 4) will be positively related to future smoking cessation behavior. Since planning is more likely to occur among smokers motivated to quit within a short period we will also assess whether the effects of specific plans will be different for smokers who are prepared to quit within one month.

## Method

Ethical approval for this study was obtained from the Medical Ethics Committee of the Academic Hospital Maastricht and Maastricht University.

### Participant recruitment and study design

For this study, a longitudinal design was used with a baseline assessment (T1) and follow-ups after one month (T2) and six months (T3). Smokers from the general population were recruited by advertisements via the website of the Dutch Smoking and Health Foundation. Smokers were asked to volunteer for a study on a new, computer-tailored, e-mail-delivered smoking cessation intervention. Participants were eligible for recruitment if they had been smokers for more than five years and were willing to quit within one year. They were informed that they had a 50% chance of enrolling in the control group and would then receive non-tailored e-mail-delivered smoking cessation information. Participants had to register and approve participation and informed consent on the project’s website. They were offered €7.50 to fill out all questionnaires. After registration, they received a login code and were asked to fill out the online baseline questionnaire. For the purpose of this study, we used the sample of the control group.

### Questionnaire

The questionnaire used for all assessments was based on a version from an earlier study [46] and assessed demographic factors, smoking behavior factors, attitudes, social influence beliefs, self-efficacy, intention, and action planning.

*Current quit attempt* was assessed by asking if participants were carrying out a quit attempt at the specific time of measurement (0 = No, 1 = Yes).

*Smoking behavior* at follow-up was assessed by asking whether participants were currently attempting to quit (0 = No, 1 = Yes) and by asking 7-day point prevalence abstinence (0 = Yes, I smoked during the past 7 days; 1 = No, I did not smoke during the past 7 days [47]). At baseline, we also assessed the number of previous attempts at quitting, years as a smoker, and number of tobacco products consumed per day, to be used as covariates.

The *demographics* assessed included age, gender, and level of education (1 = no education; primary or vocational school; 2 = secondary school; 3 = higher education, such as college/university).

*Level of cigarette dependence* was assessed by the 6-item Fagerström ([48]; 0 = no dependence, 10 = high dependence; Cronbach’s alpha = 0.70).

*Depression* was assessed by the 10-item CES-D questionnaire ([49]; 10 = no indications of depression, 40 = strong indications of depression; Cronbach’s alpha = 0.86).

*Number of quit attempts* was assessed by asking how many times the participant had tried to quit smoking previously (open ended question). Responses ranged from 1 to 16 times.

*Intention to quit* was assessed by asking when smokers intended to quit. Responses ranged from smokers intending to quit within a month (1) to smokers intending to quit within a year (0) [50].

*Attitude* was measured with two subscales. The pros of quitting consisted of the mean score of 9 statements on a 4-point scale (0 = No, certainly not; 4 = Yes, certainly; Cronbach’s alpha = 0.78). The cons of quitting were measured by nine statements on a 4-point scale (0 = No, certainly not; 4 = Yes, certainly; Cronbach’s alpha = 0.60).

*Social influence* was assessed by two questions measuring the smoking behavior of the children and/or partner (1 = smoking, 0 = not smoking). These two questions were included separately in the analyses. Having no partner or no children was coded as 0 (not smoking).

*Self-efficacy* was measured by 16 questions on a 5-point scale (1 = disagree, 5 = agree) asking participants to indicate how confident they were about overcoming specific barriers such as not smoking at parties, not smoking after dinner, etc. All scores were averaged to form a self-efficacy scale (Cronbach’s alpha = 0.90).

*Planning* was assessed by asking respondents to rate on a 5-point scale (1 = definitely not, 5 = definitely yes) if they planned to carry out each of 12 different preparatory plans. The plans were selected from earlier studies [22,42,46]. All scores were averaged to form an action-planning scale (Cronbach’s alpha = 0.68).

*Plan enactment:* At both follow-ups, participants were asked which of the 12 action plans they had executed. For each of the 12 plans, respondents could answer with Yes (1) or No (0). All scores were summed to form a plan enactment scale (Cronbach’s alpha = 0.72).

### Analysis

All analyses were done using SPSS 19. Descriptive analyses were undertaken to describe the sample, and logistic regression was used to assess predictors of dropout. To describe the percentage of preparatory plans made in Table 1, scores were dichotomized as follows: “definitely yes” and “yes” into 1 vs. “not sure,” “no,” and “definitely no” into 0. Concerning Hypotheses 1 and 2, linear regression analysis was used to explore whether individuals who intended to quit within the next month would exhibit higher levels of action planning at baseline and whether they would execute more action plans at follow-up (both normally distributed). To test additional explained variance and to add covariates, the enter method was used for all models. For Hypotheses 3 and 4, logistic regression analysis was used to explore if the levels of action planning and plan enactment were associated with smoking cessation. We used the test of joint significance [51] to asses if, and to what extent, the effect of action planning was mediated by plan enactment. Furthermore, age, education, gender, previous attempts to quit, level of cigarette dependence, level of depression, attitude, social influence, and self-efficacy were added to all models as covariates.

**Table 1:** Action plans and their enactment

	**Planned at baseline (T1)**		**Executed after 1 month (T2)**		**Executed after 6 months (T3)**	
	**Total**	**PR**	**Total**	**PR**	**Total**	**PR**
To remove all tobacco products from your house	72.5% (729)	74.6% (586)	39.1% (157)	46.3% (145)	42.7% (99)	43.9% (82)
To choose a specific quit date	82.2% (826)	87.2% (685)	50.7% (204)	56.5% (177)	58.6% (136)	59.4% (111)
To inform people in your environment that you are about to quit smoking	60.2% (605)	64.0% (503)	49.5% (199)	53.4% (167)	51.7% (120)	50.3% (94)
To do something different when you feel like smoking	76.4% (768)	76.3% (600)	44.8% (180)	48.6% (152)	53.4% (124)	54.5% (102)
To think about how you can prevent weight increase after you have quit smoking	73.8% (742)	73.9% (581)	41.8% (168)	45.4% (142)	46.6% (108)	49.7% (93)
To remove all ashtrays from the house	43.0% (432)	45.2% (355)	21.4% (86)	25.2% (79)	25.0% (58)	25.7% (48)
To search for certain aids to help you quit smoking	45.1% (454)	42.4% (333)	32.8% (132)	35.1% (110)	38.4% (89)	41.2% (77)
To break your habit of smoking at specific times and places	85.8% (863)	86.1% (677)	44.3% (178)	46.6% (146)	49.6% (115)	51.3% (96)
To read information about how to quit successfully	89.4% (898)	90.5% (711)	68.4% (275)	70.6% (221)	62.9% (146)	63.6% (119)
To think about how to cope with difficult situations before your quit attempt	74.6% (750)	75.7% (595)	33.6% (135)	34.5% (108)	39.2% (91)	38.5% (72)
To make non-smoking agreements with the individuals living with you	50.4% (50 7)	51.0% (401)	18.7% (75)	19.5% (61)	22.8% (53)	23.5% (44)
To find a buddy so you can quit together	30.3% (305)	31.4% (247)	20.9% (84)	21.7% (68)	19.4% (45)	20.9% (39)

## Results

### Sample characteristics

In total, 1,005 participants filled out the online questionnaire at baseline. Mean age was 38.8 years (*SD* = 11.6); 68% were women. Participants smoked 19.8 cigarettes per day (*SD* = 8.6), had a smoking history of 21.3 years (*SD* = 11.4), and had made 3.8 previous attempts to quit (*SD* = 5.1). Mean cigarette dependence as measured by the Fagerström questionnaire was 4.9 (*SD* = 2.5). After one month, 221 participants (55% of the complete cases) reported to be currently executing a quit attempt. After six months, 114 participants (49% of the complete cases) reported to be executing a quit attempt. Similarly, complete case analysis indicated that 124 participants had quit smoking (measured by 7-day point prevalence) after one month (34%) and 97 participants after six months (41%). When participants who were lost to follow-up are treated as smokers these percentages drop to 12% and 10%, respectively.

The sample prepared to quit within a month consisted of 786 participants, aged 38.7 years (*SD* = 11.4), mostly women (70%), who smoked 19.6 cigarettes per day (*SD* = 8.5), had a smoking history of 21.2 years (*SD* = 11.2), and had made 3.9 previous attempts to quit (*SD* = 5.3). Mean cigarette dependence was 4.9 (*SD* = 2.5). After one month, 197 participants (63% of the complete cases) reported to be currently executing a quit attempt. After six months, 95 participants (51% of the complete cases) reported to be executing a quit attempt. Complete case analysis showed that 118 participants had quit smoking (measured by 7-day point prevalence) after one month (38%) and 85 participants after six months (45%). When participants who were lost to follow-up are treated as smokers these percentages drop to 15% and 11%, respectively.

### Attrition

At follow-up after one month, 402 participants (40%) filled out the online questionnaire. Logistic regression analysis revealed that participants who dropped out after one month were more likely to be less educated (OR = 0.75, *p* < .01, 95% CI: 0.61–0.92). After six months, 232 participants (23%) filled out the web questionnaire and the only factor that predicted dropout was having a smoking partner (OR = 1.38, *p* = .05, 95% CI: 1.00–1.93).

Among smokers prepared to quit within a month, 313 participants (40%) filled out the online questionnaire after one month. Participants who dropped out after one month were more likely to be less educated (OR = 0.76, *p* < .05, 95% CI: 0.61–0.96), had a higher chance of having a partner that smokes (OR = 1.44, *p* < .05, 95% CI: 1.04-2.00), and were younger (OR = 0.97, *p* < .01, 95% CI: 0.95-0.98). After six months, 187 participants (23%) filled out the web questionnaire and factors that predicted dropout were having a partner who smokes (OR = 1.47, *p* < .05, 95% CI: 1.01–2.15) and age (OR = 0.97, *p* < .01, 95% CI: 0.95-0.99)

### Plans and their enactment

Our first goal pertains to the analysis of plans chosen and executed. As can be noted from Table 1, the most popular plans for the total population at baseline were to read information about how to quit successfully (89.4%), to break the habit of smoking at specific times and places (85.8%), and to choose a specific date to quit (82.2%). At follow-up one month later (T2), we assessed which of the 12 plans participants had executed. Results reveal that reading information about how to quit successfully was the plan that was executed most (68.4% of the complete cases). Informing people in the environment that you are about to quit smoking and choosing a specific date to quit were plans executed by roughly half of the participants (49.5% and 50.7%, respectively). At the second follow-up six months after baseline (T3), a similar pattern was observed, with reading information about how to quit successfully still being the most executed plan (62.9%), followed by choosing a specific date to quit (58.6%), and doing something different when you feel like smoking (53.4%). Making non-smoking agreements and finding a buddy were the least popular plans and were carried out by only a fifth of the participants (T2 and T3). As can be noted, these figures were only marginally different for smokers who were prepared to quit within a month.

### Effects of specific preparatory plans and plan enactment

The second goal of the study aims at assessing which plans were related to attempts to quit and smoking cessation. Table 2 shows the results of the univariate logistic regression analyses, in which attempts to quit and actual smoking cessation were regressed on each plan separately. The results reveal that associations of plan enactment with quit attempt at T2 and smoking cessation at T2 and T3 were stronger than the association of action planning, both for the total population and for smokers prepared to quit within one month.

**Table 2:** Action plans and goal enactment predicting quit attempt and smoking cessation (Univariate analyses)

		**Quit attempt (T2)**		**7 day point prevalence (T2)**		**7 day point prevalence (T3)**	
		**OR**_**TOT**_	**OR**_**PR**_	**OR**_**TOT**_	**OR**_**PR**_	**OR**_**TOT**_	**OR**_**PR**_
To remove all tobacco products from your house	AP	1.09	1.12	1.34^**^	1.37^**^	1.07	1.09
	PE	3.59^***^	2.59^***^	4.50^***^	2.94^***^	3.99^***^	3.44^***^
To choose a specific quit date	AP	1.59^***^	1.29^*^	1.77^***^	1.38^*^	1.33^*^	1.13
	PE	3.02^***^	2.26^**^	3.07^***^	2.55^***^	3.79^***^	3.96^***^
To inform people in your environment that you are about to quit smoking	AP	1.27^**^	1.23^*^	1.45^***^	1.34^**^	1.18^†^	1.18
	PE	2.63^***^	2.72^***^	2.31^***^	1.95^**^	2.35^**^	2.46^**^
To do something different when you feel like smoking	AP	1.17	1.08	1.14	1.17	1.02	0.96
	PE	3.12^***^	2.82^***^	2.52^***^	2.01^**^	2.42^**^	1.96^*^
To think about how you can prevent weight increase after you have quit smoking	AP	0.97	0.94	1.13	1.17	1.12	1.13
	PE	1.77^**^	1.45	1.70^*^	1.51^†^	2.03^**^	1.79^*^
To remove all ashtrays from the house	AP	1.06	1.05	1.13^†^	1.10	1.08	1.06
	PE	2.08^**^	1.48	1.54^†^	1.17	2.50^**^	2.08^*^
To search for certain aids to help you quit smoking	AP	1.06	1.10	1.02	1.08	0.89	0.95
	PE	2.27^***^	2.09^**^	1.98^**^	1.86^*^	1.33	1.09
To break your habit of smoking at specific times and places	AP	1.40^**^	1.22	1.16	1.06	1.25	1.45^†^
	PE	1.58^*^	1.25	1.21	1.05	2.74^***^	2.73^**^
To read information about how to quit successfully	AP	1.47^**^	1.40^*^	1.37^*^	1.29	0.94	0.78
	PE	1.58^*^	1.21	1.26	1.19	1.58	1.75^†^
To think about how to cope with difficult situations before your quit attempt	AP	1.17	1.01	1.07	0.91	0.97	0.85
	PE	1.36	1.36	1.46^†^	1.45	1.55	1.48
To make non-smoking agreements with the individuals living with you	AP	0.96	0.99	1.05	1.13	0.98	1.08
	PE	1.29	1.26	1.15	1.09	1.62	1.61
To find a buddy so you can quit together	AP	0.95	0.89	0.98	1.03	0.97	1.04
	PE	0.88	0.59^†^	1.01	0.87	1.14	1.04

*AP* = Action Plans; *PE* = Plan Enactment; † *p* < .10, * *p* < .05, ** *p* < .01, *** *p* < .001.

Total sample: *N* (T2) = 402; *N* (T3) = 232; PR sample: *N* (T2) = 313; *N* (T3) = 187.

Table 2, furthermore, reveals that enactment of the six following plans is associated with increased quit attempt rates at T2 and smoking cessation at T2 and T3 for both groups: removing all tobacco products from your house, choosing a specific quit date, informing people in your environment that you are going to quit, doing something different when you feel like smoking, thinking about preventing weight increase, removing all ash trays, and breaking your habit of smoking at specific times and places.

Table 3 shows the results of the multivariate logistic regression analyses (with age, gender, education, self-efficacy, cigarette dependence, and intention to quit as covariates), in which all plan enactments were included. VIF (all < 2) and tolerance values (all > 0.5) revealed no threats for multicollinearity between the items and power analysis indicated that, despite our relatively small sample size of 187 participants at the second follow-up (T3), there was sufficient power to conduct the analyses (Total sample: *N* = 232, observed *R*^2^ = 0.36, power = 0.99; PR sample: *N* = 187, observed *R*^2^ = 0.33, power = 0.99; [52]).

**Table 3:** Action plans and goal enactment predicting quit attempt and smoking cessation (Multivariate analyses)

		**Quit attempt (T2)**		**7 day point prevalence (T2)**		**7 day point prevalence (T3)**	
		**OR**_**TOT**_	**OR**_**PR**_	**OR**_**TOT**_	**OR**_**PR**_	**OR**_**TOT**_	**OR**_**PR**_
To remove all tobacco products from your house	AP	0.93	1.00	1.25^†^	1.24^*^	1.08	0.92
	PE	1.70^†^	1.80^†^	3.55^***^	2.96^**^	3.40^**^	3.56^**^
To choose a specific quit date	AP	1.25^†^	1.22	1.24	1.22	1.03	1.09
	PE	1.88^*^	1.62^†^	1.95^*^	2.06^*^	3.34^***^	3.65^**^
To inform people in your environment that you are about to quit smoking	AP	1.19^†^	1.23^†^	1.30^*^	1.26^*^	1.16	1.14
	PE	1.75^*^	2.48^**^	1.24	1.21	1.47	1.59
To do something different when you feel like smoking	AP	1.02	0.98	1.14	1.02	0.89	0.98
	PE	2.44^**^	2.74^**^	1.94^*^	1.75^*^	1.31	0.89
To think about how you can prevent weight increase after you have quit smoking	AP	0.91	0.94	1.10	1.20^†^	1.20	1.18
	PE	1.58^†^	1.40	1.40	1.36	1.86^†^	1.53
To remove all ashtrays from the house	AP	1.01	1.07	0.98	1.04	1.06	1.11
	PE	0.95	0.89	0.54^†^	0.57^†^	0.94	0.71
To search for certain aids to help you quit smoking	AP	1.10	1.08	1.08	1.02	0.99	0.93
	PE	1.63^†^	1.80^†^	1.37	1.45	0.94	0.82
To break your habit of smoking at specific times and places	AP	1.18	1.09	0.86	0.84	1.45^†^	1.09
	PE	0.87	0.68	0.63	0.63	1.71	2.30^†^
To read information about how to quit successfully	AP	1.43^*^	1.50^*^	1.48^*^	1.17	0.80	0.91
	PE	0.94	0.69	0.79	0.85	0.96	1.18
To think about how to cope with difficult situations before your quit attempt	AP	1.01	0.87	0.79^†^	0.85	0.88	0.81^†^
	PE	0.76	0.76	1.07	1.07	0.77	0.70
To make non-smoking agreements with the individuals living with you	AP	0.88	0.91	0.98	0.94	0.91	0.95
	PE	0.94	1.32	0.81	0.86	0.72	0.89
To find a buddy so you can quit together	AP	0.96	0.88	0.97	0.94	0.96	0.98
	PE	0.55^†^	0.29^**^	0.66	0.59	0.73	0.66

*AP* action plans; *PE* plan enactment; † *p* < .10, * *p* < .05, ** *p* < .01, *** *p* < .001.

Total sample: *N* (T2) = 402; *N* (T3) = 232; PR sample: *N* (T2) = 313; *N* (T3) = 187.

The results of Table 3 confirm the pattern identified in Table 2, that making plans is less predictive of smoking cessation behavior than plan enactment. Most Odds Ratios were considerably higher for plan enactment than for making plans. Many enacted plans that predicted quit attempt and smoking cessation had a comparable strength both for the total population and smokers prepared to quit within one month. As can be noted, the differences in the ORs between these two groups were mostly marginal.

Finally, two plan enactments predicted smoking cessation consistently at both follow-ups: removing all tobacco products from the house, and choosing a specific date to quit. Table 3, furthermore, reveals that finding a buddy with whom to quit, making non-smoking agreements, and thinking about how to cope with difficult situations before attempting to quit were relatively ineffective plans. They had no significant association with any of the three outcomes for the total population. Moreover, finding a buddy with whom to quit was found to be negatively associated with quit attempt rates (T2) for smokers prepared to quit within a month.

### Intention is related to action plan development

Next, we assessed which factors were associated with making action plans. As suggested by Hypothesis 1, we found higher levels of action planning in smokers who intended to quit within the next month (*M* = 3.80, *SD* = 0.54) than in those intending to quit within a year (*M* = 3.60, *SD* = 0.55; *t*(1003) = 4.77, *p* < .001). Similarly, participants who intended to quit smoking within the next month had executed significantly more plans at follow-up one month later (*M* = 5.04, *SD* = 2.71) than those intending to quit within a year (*M* = 3.34, *SD* = 2.56; *t*(400) = 5.28, *p* < .001). Multivariate linear regression analysis was used to assess which other factors were related to action planning. Besides the effect of intention, the results revealed that being older, being female, having relatively high levels of cigarette dependence, perceiving more positive and negative consequences (pros and cons) of quitting, and high self-efficacy toward quitting were all associated with higher levels of action planning at baseline (Table 4).

**Table 4:** Baseline variables associated with action planning and plan enactment

	**Action planning (T1)**	**Executed plans (T2)**	**Executed plans (T3)**
	***β***	***β***	***β***
Total Sample			
Age	.10^***^	-.12^*^	-.07
Gender (0 = male)	.07^**^	.04	.09
Education	.04^†^	.01	.11
Depression	.01	.07	.05
Quit attempts	-.01	.05	.10
Nicotine dep.	.11^***^	.07^†^	.05
Pros of quitting	.29^***^	.03	.06
Cons of quitting	.08^**^	.05	.10
SI – children	-.02	.02	.01
SI – partner	.01	.01	-.03
Self-efficacy	.18^***^	.01	.05
Intention to quit	.07^**^	.15^**^	-.01
Action planning	-	.37^***^	.32^***^
Smokers In Preparation			
Age	.10^**^	-.13^*^	-.01
Gender (0 = male)	.10^*^	.08	.12
Education	.06^†^	.03	.18^*^
Depression	.02	.06	.06
Quit attempts	-.03	.07	.07
Nicotine dep.	.10^**^	.06	.03
Pros of quitting	.29^***^	.03	.09
Cons of quitting	.06	.07	.13
SI – children	-.01	-.01	-.01
SI – partner	.01	-.05	-.05
Self-efficacy	.22^***^	-.01	.05
Action planning	-	.37^***^	.27^**^

*SI* = Social influence; † *p* < .10, * *p* < .05, ** *p* < .01, *** *p* < .001.

### Intention is related to plan enactment

As suggested by Hypothesis 2a, the multivariate linear regression analyses depicted in Table 4 also reveal that intention was positively related to the number of plans executed at T2. In addition, younger participants and participants who reported higher levels of action planning at baseline (as suggested by Hypothesis 2b) executed more plans. After six months (T3), the effects of intention and age dissipated; action planning at baseline was the only predictor of plan execution.

### Action planning and plan enactment are related to smoking cessation

Hypotheses 3 and 4 postulate that action planning and plan enactment are related to smoking cessation. The multivariate logistic regression analyses in Table 5 reveal that the level of action planning at baseline only predicted smoking cessation for smokers motivated to quit within a month at the first follow-up, and had only a borderline significant effect on smoking cessation for the total population at the first follow-up. Plan enactment, however, consistently predicted smoking cessation for both groups, as suggested by Hypothesis 4. Other predictors of non-smoking after six months for the total sample were low levels of depression, having a non-smoking partner, and intending to quit within the next month. Low levels of cigarette dependence and high self-efficacy to quit were borderline significant predictors. Other factors predicting nonsmoking after six months for smokers motivated to quit were depression and self-efficacy.

**Table 5:** Factors associated with quit attempt and smoking cessation

	**Quit attempt (T2)**		**7 day point prevalence (T2)**		**7 day point prevalence (T3)**	
	**OR**_**TOT**_	**OR**_**PR**_	**OR**_**TOT**_	**OR**_**PR**_	**OR**_**TOT**_	**OR**_**PR**_
Age	1.02	1.02	1.01	1.01	1.01	1.01
Gender (0 = male)	0.41^*^	0.42^*^	0.65	0.69	0.76	0.54
Education	0.99	1.05	0.99	1.01	0.82	0.86
Depression	0.97	0.97	0.95	0.94^†^	0.92^*^	0.89^*^
Quit attempts	0.98	0.97	0.99	0.99	0.95	0.95
Nicotine dep.	0.94	0.91	0.92	0.93	0.87^†^	0.90
Pros of quitting	1.12	1.32	0.73	0.73	0.74	0.77
Cons of quitting	1.02	0.93	0.76	0.78	0.86	0.84
Smoking children	1.20	1.49	1.05	1.09	0.65	0.81
Smoking partner	0.83	0.67	0.67	0.63^†^	0.45^*^	0.56
Self-efficacy	1.39	1.49	1.49	1.52	1.89^†^	2.36^*^
Intention to quit	3.86^***^	-	6.83^***^	-	2.60^*^	-
AP at baseline	0.88	0.87	1.76^†^	1.90^*^	0.72	0.71
PE after 1 month	1.30^***^	1.27^***^	1.22^***^	1.20^**^	-	-
PE after 6 months	-	-	-	-	1.50^*^	1.48^***^

*AP* = Action Plans; *PE* = Plan Enactment; † *p* < .10, * *p* < .05, ** *p* < .01, *** *p* < .001.

Total sample: *N* (T2) = 402; *N* (T3) = 232; PR sample: *N* (T2) = 313; *N* (T3) = 187.

Furthermore, we tested potential mediation effects in order to explore how much of the predictive value of action planning could be attributed to the actual execution of such a plan [53]. Results reveal that 81% of the direct effect of action planning was mediated by plan enactment. Using the Sobel test, this mediation was found to be significant (*t* = 1.95, *p* < .05; [54]).

Finally, we translated the effects of plan enactment into actual quitting percentages by creating two groups: participants low in plan enactment (less than the median of five after six months) and participants high in plan enactment (more than the median of five after six months). This revealed that 29% of the smokers who completed less plans than the median had quit successfully after six months versus 59% of the smokers who completed more than the median (complete case analysis; *N* = 232; χ^2^ = 21.05, *p* < .001).

Similarly, participants who intended to quit smoking within the next month had executed significantly more plans at follow-up one month later (*M* = 5.04, *SD* = 2.71) than those intending to quit within a year (*M* = 3.34, *SD* = 2.56; *t*(400) = 5.28, *p* < .001).

## Discussion

Planning can have a significant positive impact on changing health behavior [55-59]. However, relatively little is known about which factors are related to action planning and plan enactment, and how they are related to smoking cessation. Our results suggest seven plans that were related to smoking cessation at follow-up after six months. The multivariate analysis identified that the execution of two specific plans was most significantly related to abstinence at follow-up, namely removing all tobacco products from the house and choosing a specific date to quit. The latter finding confirms the previous work of Balmford and colleagues [60]. Yet, it is conceivable that the five plans that were not significant anymore in the multivariate analysis are related to these two plans and that for selection of effective plans to quit smoking, a practitioner might want to choose to recommend these seven plans. In addition, we identified plans that were not related to smoking cessation after one and six months. They included finding a buddy with whom to quit, making non-smoking agreements, and thinking about how to cope with difficult situations before attempting to quit. The latter finding is in contrast to other notions of the importance of coping plans. One explanation is that this type of formulation may have been too broad and may need to be replaced by specific implementation intentions for specific difficult situations.

As suggested by Hypothesis 1, higher levels of action planning were observed in smokers who intended to quit within the next month. Additional factors that correlated with baseline action planning were being older, being female, high levels of cigarette dependence, perceiving more positive consequences (pros) of quitting, perceiving more negative consequences (cons) of quitting, and having high self-efficacy toward quitting. Although the importance of these factors for predicting successful quitting in (subgroups of) smokers has been documented by several other studies (see e.g., [30,61-64]), their relation to the process of action planning was not assessed.

In line with Hypothesis 2, our results reveal that the intention to quit and action planning at baseline had a significant and positive influence on the number of plans executed. Other studies have already noted that action planning will be more likely to occur under high levels of motivation [5,17,58], but not that these factors are also related to plan enactment. Participants who intended to quit smoking within the next month had executed, on average, two and a half more action plans at follow-up one month later than their peers who intended to quit within a year. These findings are confirmed by a recent study that found the greatest effect of preparatory planning among smokers who tried to quit by following a group course [65]. At the six-month follow-up, the effect of intention was no longer significant, and action planning at baseline was the only significant predictor of plan enactment.

We found support for Hypothesis 3 stating that planning is associated with quit attempts and smoking cessation. Several plans have been found to be associated with quit attempts and smoking cessation. Yet, when controlling for other factors we noted that the general factor of planning was only a significant predictor of smoking cessation at first follow-up and only for smokers in preparation. We also found support for Hypothesis 4, postulating that plan enactment would significantly predict non-smoking status at follow-up. Other significant predictors of smoking cessation were depression, and self-efficacy. Of interest is the fact that our findings do not strongly support studies that suggest an important role for cigarette addiction in understanding cessation maintenance [66,67]. We only found a borderline significant effect of cigarette dependence in predicting smoking cessation in the total population at the last-follow up.

Another finding was that baseline action planning was not a significant independent predictor of cessation outcome after controlling for other factors, including plan enactment. These results suggest that the effects of action planning may be mediated by plan enactment. We indeed found support for this mediation effect, since our results estimated that 81% of the direct effect of action planning was mediated by plan enactment. Furthermore, our results revealed that 29% of the smokers who completed fewer plans than the median had quit successfully after six months versus 59% of the smokers who completed more than the median. Action planning is an important precondition for plan enactment, because planning is obviously more likely to lead to enactment of certain goals. Plan enactment after one month was predicted by baseline intentions and baseline action planning and, after six months, only by action planning. This last finding illustrates the importance of translating global baseline intentions into specific plans.

Comparing the findings concerning the plans made and executed between our total population with those prepared to quit within one month showed similarity in the plans made and executed. A notable difference was the significance of breaking habits at the last follow-up, which appeared to be a significant predictor of smoking cessation among those prepared to quit. This finding supports other research that outlines the impact of habits on behavior [68], but even the importance of planning to break habits. We also found that smokers motivated to quit within one month had executed more plans. This suggests that adopting and executing effective plans can be productive to aid smoking cessation. This is supported by our finding showing that those who completed more plans than the medium were also more effective in remaining non-smokers after six months.

Our study is subject to some limitations. First, we did not assess differences in levels of specificity of the action plans. Van Osch and colleagues [16] found that plan specificity positively predicts point prevalence abstinence; medium to highly specific planning resulted in higher abstinence rates than less specific planning. Hence, assessing levels of specificity in future studies is recommended. Second, we did not make two separate scales of preparatory plans and coping plans in order to assess a potential different impact of both types of plans. Sniehotta, 2005 [13]; Sniehotta, 2009 [14]; de Vet, 2011 [34], nor did we assess the exact timing of plan enactment (e.g. before or after the quit attempt). Future studies should also assess the role of both types of planning [69] and assess the potential differential impact of preparatory and coping plan enactment. Third, our study had low retention rates at follow-up. However, because only low educational level and having a partner that smokes were related to dropout, the likelihood of a bias that those who quit smoking were more likely to provide follow-up data is small.

## Conclusion

Our results reveal clearly the importance of the assessment of both action planning and plan enactment, factors that were also identified in a recent review of behavior-change strategies for smoking cessation [70]. Application of these behavior change strategies can be used by various methods such as mass-media campaigns, health counseling, and computer tailoring [16,62,71-73]. Our results support notions identified by others, such as Zhou and colleagues [74] indicating that motivational factors may be relevant for predicting smoking cessation attempts, but that other factors are also involved when predicting smoking cessation maintenance. Future studies about action planning should not only assess which plans are made but also which plans are enacted. Our study reveals that smoking cessation is more likely to be successful when effective action plans are both formulated and executed. Our study also suggests that not all action plans are necessarily predictive of effective smoking cessation. Haug and colleagues [64], for instance, found that some constructs of the Transtheoretical model (i.e., helping relationships, commitment to change, coping with temptations) were univariately related to smoking cessation, but did not have a unique prediction in a more comprehensive model. This illustrates the importance of longitudinal and experimental research as well as the use of a comprehensive model to identify which factors predict smoking cessation. Furthermore, it is relevant to study differences in decision-making for volitionally chosen and assigned action plans [45]. Although participation in goal setting may increase goal commitment [75], it is also conceivable that participants may choose ineffective action plans, as can be seen from the results in our study. Yet, it may be likely that a goal chosen with a high degree of confidence in the decision-making procedure used is more likely to be enacted than one fraught with uncertainty [76]. This suggests that action planning and levels of self-efficacy toward planning enactment may have interactive effects. Finally, some studies suggest the importance of unplanned attempts to quit, but cannot really exclude whether or not action plans were not made, and used retrospective designs (see e.g., [77-79]). Other studies do not support these claims (see e.g., [80,81]). Our study clearly demonstrates that discussions about whether or not to use planning need better documentation than has been made in the past, namely by a clear assessment of plans made and enacted.

## Competing interests

HdV is also scientific director of Vision2Health, a company implementing evidence based eHealth programs.

## Authors’ contribution

HdV conceived the study, supervised the development and implementation of the study, and drafted the manuscript. ME performed the statistical analyses and participated in the drafting of the manuscript. CB was participated in the development and implementation of the study and the drafting of the manuscript. All authors read and approved the final manuscript.

## Authors’ information

HdV is professor in health communication at Maastricht University with a special interest in testing social cognitive models, designing computer tailored eHealth interventions, and various other types of health communications for various topics. ME is researcher in health communication at Maastricht University with special interests in theory building and testing and HIV prevention. CB is assistant professor in health psychology with special interests in smoking cessation.

## Pre-publication history

The pre-publication history for this paper can be accessed here:

http://www.biomedcentral.com/1471-2458/13/393/prepub
